# Bactericidal Immunity to *Salmonella* in Africans and Mechanisms Causing Its Failure in HIV Infection

**DOI:** 10.1371/journal.pntd.0004604

**Published:** 2016-04-08

**Authors:** Yun Shan Goh, Francesca Necchi, Colette M. O’Shaughnessy, Francesca Micoli, Massimiliano Gavini, Stephen P. Young, Chisomo L. Msefula, Esther N. Gondwe, Wilson L. Mandala, Melita A. Gordon, Allan J. Saul, Calman A. MacLennan

**Affiliations:** 1 Singapore Immunology Network, Agency for Science, Technology and Research, Biopolis, Singapore; 2 Sclavo Behring Vaccines Institute for Global Health, a GlaxoSmith Kline Company, Siena, Italy; 3 School of Immunity and Infection, College of Medicine and Dental Sciences, University of Birmingham, Birmingham, United Kingdom; 4 GlaxoSmith Kline, Siena, Italy; 5 Centre for Translational Inflammation Research, College of Medical and Dental Sciences, University of Birmingham, Birmingham, United Kingdom; 6 Malawi-Liverpool-Wellcome Trust Clinical Research Programme, College of Medicine, University of Malawi, Blantyre, Malawi; 7 Department of Pathology, Division of Microbiology, College of Medicine, University of Malawi, Blantyre, Malawi; 8 Department of Basic Medical Sciences, College of Medicine, University of Malawi, Blantyre, Malawi; 9 Department of Gastroenterology, Institute of Translational Medicine, University of Liverpool, Liverpool, United Kingdom; 10 Jenner Institute, Nuffield Department of Medicine, University of Oxford, Oxford, United Kingdom; University of California San Diego School of Medicine, UNITED STATES

## Abstract

**Background:**

Nontyphoidal strains of *Salmonella* are a leading cause of death among HIV-infected Africans. Antibody-induced complement-mediated killing protects healthy Africans against *Salmonella*, but increased levels of anti-lipopolysaccharide (LPS) antibodies in some HIV-infected African adults block this killing. The objective was to understand how these high levels of anti-LPS antibodies interfere with the killing of *Salmonella*.

**Methodology/Principal Findings:**

Sera and affinity-purified antibodies from African HIV-infected adults that failed to kill invasive *S*. Typhimurium D23580 were compared to sera from HIV-uninfected and HIV-infected subjects with bactericidal activity. The failure of sera from certain HIV-infected subjects to kill *Salmonella* was found to be due to an inherent inhibitory effect of anti-LPS antibodies. This inhibition was concentration-dependent and strongly associated with IgA and IgG2 anti-LPS antibodies (p<0.0001 for both). IgG anti-LPS antibodies, from sera of HIV-infected individuals that inhibit killing at high concentration, induced killing when diluted. Conversely, IgG, from sera of HIV-uninfected adults that induce killing, inhibited killing when concentrated. IgM anti-LPS antibodies from all subjects also induced *Salmonella* killing. Finally, the inhibitory effect of high concentrations of anti-LPS antibodies is seen with IgM as well as IgG and IgA. No correlation was found between affinity or avidity, or complement deposition or consumption, and inhibition of killing.

**Conclusion/Significance:**

IgG and IgM classes of anti-*S*. Typhimurium LPS antibodies from HIV-infected and HIV-uninfected individuals are bactericidal, while at very high concentrations, anti-LPS antibodies of all classes inhibit in vitro killing of *Salmonella*. This could be due to a variety of mechanisms relating to the poor ability of IgA and IgG2 to activate complement, and deposition of complement at sites where it cannot insert in the bacterial membrane. Vaccine trials are required to understand the significance of lack of in vitro killing by anti-LPS antibodies from a minority of HIV-infected individuals with impaired immune homeostasis.

## Author Summary

Bacteremia caused by nontyphoidal *Salmonellae* are a major health burden in Africa. While antibody-induced complement-mediated killing protects healthy Africans against *Salmonella*, increased levels of anti-LPS antibodies in some HIV-infected Africans block this killing. Little is known about the mechanism of the interference of killing by these antibodies. Here, we compared sera and affinity-purified antibodies from African HIV-infected adults that are unable to kill invasive *S*. Typhimurium D23580, with sera from HIV-uninfected and HIV-infected subjects with bactericidal activity. We found that the blocking effect of anti-LPS antibodies is a factor of antibody concentration, rather than antibody structure or specificity. While all three isotypes (IgG, IgA and IgM) can inhibit killing of *Salmonella* at grossly high concentrations, the IgG and IgM isotypes of the anti-LPS antibodies have in vitro bactericidal activity against invasive African *S*. Typhimurium. Inhibition of killing did not associate with antibody affinity or avidity, or complement deposition or consumption. It is possible that a LPS-based vaccine would induce antibodies at bactericidal rather than inhibitory concentrations in HIV-uninfected individuals. In HIV-infected individuals, it is uncertain whether vaccination will induce a protective response or a dysregulated excess of anti-LPS antibodies that impairs serum killing of *Salmonella*.

## Introduction

African clades of nontyphoidal *Salmonella* (NTS), particularly *S*. *enterica* serovars Typhimurium and Enteritidis, are a major cause of bacteremia in sub-Saharan Africa [1, 2]. Case fatality rates are around 20–25% [1], and up to 47% in HIV-infected adults [3] prior to the availability of antiretroviral therapy. Diagnosing NTS bacteremia is difficult due to a lack of specific clinical presentation. The emergence of multi-drug resistant isolates [4] has added to the problem of management and no vaccine is currently available. NTS bacteremia in Africa occurs most frequently among infants and HIV-infected patients [1, 2]. The underlying mechanisms of susceptibility are not fully understood. We have previously shown that sera from African children under two years of age lack *Salmonella*-specific antibodies, resulting in an impaired ability to kill *Salmonella*. Sera from adults with *Salmonella*-specific antibodies can induce complement-mediated killing of *Salmonella* and placental transfer of IgG offers protection to infants [5], suggesting a role for antibody in protection against invasive NTS (iNTS) disease.

Mice can be protected against an intraperitoneal challenge with *S*. Typhimurium either by immunization with experimental LPS O-antigen-based conjugate vaccines [6, 7] or passive transfer of monoclonal antibodies against O-antigens [8, 9]. The role of anti-LPS antibodies in protection against iNTS disease in man is not fully appreciated, although antibodies to *S*. Typhimurium LPS O-antigen correlate with serum killing of *S*. Typhimurium D23580 in Malawian children [10].

Immunity against iNTS in HIV-infected adults is complex. Our work in Malawi demonstrates an association between impaired serum killing of NTS and dysregulated production of high levels of antibodies to *S*. Typhimurium LPS in some HIV-infected African adults [11]. Removal of LPS-specific antibodies restores bactericidal activity. In this study, we investigate the mechanism of interference with killing of NTS by antibodies to LPS O-antigen in HIV-infected African adults. This is important for understanding the potential effectiveness of an NTS O-antigen-based vaccine in Africa, particularly in the context of HIV infection.

## Methods

### Sera

Sera were from HIV-infected and HIV-uninfected Malawian adults (S1 Table) and were the same as previously studied [11]. No individuals had a known clinical history of iNTS disease. The study was approved by the College of Medical Research and Ethics Committee, College of Medicine, University of Malawi. Written informed consent was obtained from participants prior to inclusion in the study.

### Bacteria

Invasive African *S*. Typhimurium D23580 belonging to the ST313 pathovar [5, 12], D23580 *galE*^*-*^, *S*. Typhimurium LT2 [13], *S*. Enteritidis D24854, *S*. Enteritidis SL7488 [14], *S*. Senftenberg 20050439 and *S*. Agona 20071186 [15] were used.

### Anti-*Salmonella* antibody binding

This was as described previously [5]. Briefly, bacteria were mixed with 10% serum (final *Salmonella* concentration 2 × 10^8^ CFU/ml). After washing, bound antibodies were detected with FITC-conjugated anti-human IgG, IgA, and IgM antibodies (Sigma-Aldrich, Milan, Italy). FL1 channel fluorescence indicates anti-*Salmonella* antibody binding.

### Anti-LPS ELISA

*S*. Typhimurium LPS (Alexis Biochemicals, Vinci, Italy) was coated onto ELISA plates at 5 μg/ml and dilutions of serum sample [10] added. Anti-LPS antibodies were detected using alkaline-phosphatase-conjugated anti-human isotype-specific antibodies (Southern Biotech, Milan, Italy).

### Serum bactericidal assays

These were as previously described [11, 16]. For SBA involving endogenous complement, bacteria in log-growth phase were added to undiluted serum (final *Salmonella* concentration 10^6^ CFU/ml) and incubated at 37°C. Viable *Salmonellae* were determined after 180 min. For SBA involving exogenous complement, bacteria were added to a mixture of heat-inactivated test serum (56^°^C for 30 minutes) and 75% baby rabbit serum (BRS, AbD Serotec, Kidlington, UK). For SBA testing inhibition of serum bactericidal activity, bacteria were added to a mixture of the purified antibodies and 50% normal human adult serum. Non-*Salmonella*-specific isotype-matched control antibodies were purified paraproteins.

### Affinity-purification of isotype-specific total antibodies

To obtain total antibody of each isotype (IgG, IgA, IgM), serum was incubated sequentially with combinations of human IgA and IgM affinity matrices (CaptureSelect, Leiden, Netherlands) and protein G affinity matrix (GE Healthcare) to remove IgA, IgM and IgG respectively. Resulting isotype-specific antibodies preparations were dialyzed against PBS.

### Extraction of anti-*Salmonella* LPS isotypes

Anti-*Salmonella* LPS antibodies were extracted from affinity-purified total IgG, IgA and IgM using a *S*. Typhimurium D23580 LPS O-antigen column, as described previously [17]. Anti-LPS antibodies were eluted with 0.1 M glycine pH 3 and neutralized with 1 M Tris-HCl pH 8.0. Extracted antibodies were dialyzed against PBS.

### k_d_ analysis

Binding of serum antibodies to *S*. Typhimurium LPS were assessed using a Biacore 3000 system (GE Healthcare). A hydrophobic HPA sensor chip was coated by passing 1 mg/ml LPS across the chip surface for 30 min at 2 μl/min, washed with 0.1 M hydrochloride acid and blocked with 0.1 mg/ml bovine serum albumin. Sera were diluted 1:2 and passed across the chip surface for 10 min at 5 μl/min. *k*_*d*_ values were calculated by fitting the binding curves to a best-fit Langmuir 1:1 model using BiaEvaluation.

### Affinity of anti-*Salmonella* LPS antibodies

ELISA plates were coated with *S*. Typhimurium LPS at either a non-limiting concentration of 5 μg/ml or limiting concentration of 0.5 μg/ml. Diluted human sera were added to both plate types and incubated with alkaline-phosphatase-conjugated anti-human IgG, IgA or IgM (Southern Biotech), then SigmaFast. Affinity was calculated as the ratio of the antibody titer with limiting plates to titer with non-limiting plates [18].

### Avidity of anti-*Salmonella* LPS antibodies

Plates were coated with *S*. Typhimurium LPS at 5 μg/ml and diluted human serum added. Half the wells were washed with 6 M urea and half with PBS-0.05% Tween 20, then incubated with secondary antibodies followed by SigmaFast, as above. The avidity index is the antibody titer in the presence of urea as a percentage of titer in the absence of urea [19].

### Absolute quantification of anti-*S*. Typhimurium LPS antibody concentrations

Anti-*S*. Typhimurium LPS antibody concentrations were determined by ELISA using control antibodies of known concentration. Plates were coated with goat anti-human IgA, IgG or IgM antibodies (Sigma-Aldrich) at 5 μg/ml. Purified anti-LPS antibody eluates, together with the control antibodies, were added in step-wise dilutions. Bound antibodies were detected using secondary antibodies, then SigmaFast, as above.

### C3/C5b-9 complement deposition assays

These were by flow cytometry as previously described [5]. Bacteria in log-growth phase were mixed with undiluted serum (final concentration 2 × 10^8^ CFU/ml), then FITC-conjugated mouse anti-C3 antibody or mouse anti-C5b-9 antibody followed by FITC-conjugated anti-mouse immunoglobulin. The bacteria were first gated on FSC and SSC to exclude bacterial cell debris. GMFI in the FL1 channel was used to indicate C3 and C5b-9 deposition.

### Functional complement assays

Total complement activity and alternative hemolytic complement activity were measured by radial immunodiffusion assays according to manufacturer’s instructions (Binding Site, Grassobio, Italy).

### Statistical methods

Spearman rank was used for estimation of correlation. Comparisons of data from different groups of sera were performed by Mann-Whitney *U*-test.

## Results

### Anti-LPS isotypes from HIV-infected adults that induce or inhibit *S*. Typhimurium killing

We previously reported that ability of serum from HIV-infected Africans to induce complement-mediated killing of *S***.** Typhimurium correlates inversely with concentration of anti-LPS antibody [11]. When *S***.** Typhimurium D23580 is cultured with serum from HIV-uninfected healthy adults, the number of viable bacteria falls to between 10% and 1% of the starting value after 180 minutes. By contrast, sera from HIV-infected adults with high anti-LPS titers fail to kill D23580.

**Fig 1:**
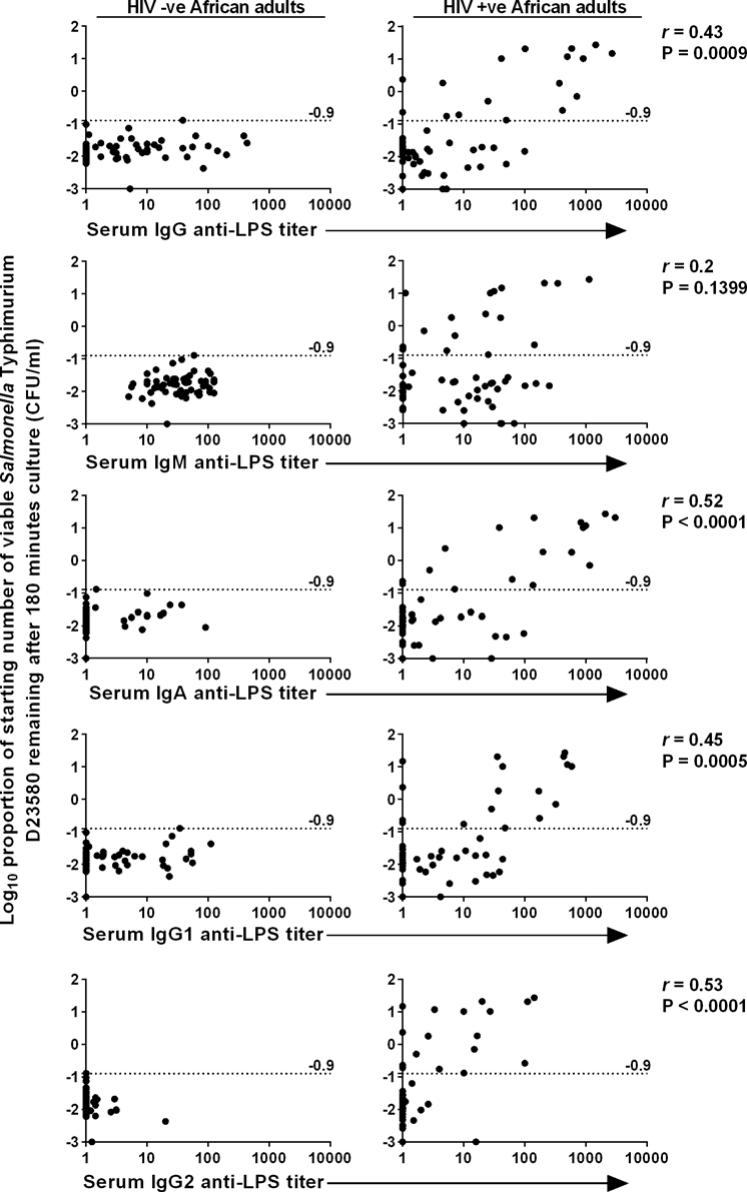
Association of impaired serum killing of *S*. Typhimurium with anti-LPS IgG, IgM, IgA, IgG1 and IgG2. Log growth phase *S*. Typhimurium D23580 was added to whole serum and the log_10_ proportion of the starting number of bacteria remaining after 180 minutes was determined on LB agar. Each panel indicates the proportion of bacteria recovered after 180 minutes in serum from HIV-uninfected subjects (n = 58, left) and HIV-infected subjects (n = 58, right) plotted against the anti-LPS concentration of that serum. Dashed line indicates threshold for impaired killing. Anti-LPS antibody concentration of the sera were determined using anti-LPS ELISA, with *S*. Typhimurium LPS coated onto ELISA plates at 5 μg/ml. Anti-LPS antibodies were detected using alkaline-phosphatase-conjugated anti-human isotype-specific antibodies. Each point represents one serum. *r*, Spearman’s correlation coefficient.

Our first objective was to test whether anti-LPS antibodies of a particular class or subclass fail to induce killing of *S***.** Typhimurium D23580 and act as a competitive inhibitor of antibodies that induce killing. As a preliminary, we assessed which serum immunoglobulin classes and subclasses are represented among anti-LPS antibodies found in HIV-uninfected and HIV-infected African adults. The ability of each individual serum to kill *S*. Typhimurium D23580 compared to IgG, IgM, IgA, IgG1 and IgG2 anti-LPS concentration of that serum is shown in Fig 1. There is a trend towards negative correlation between serum killing capacity and anti-LPS levels of each antibody isotype that only fails to reach statistical significance for IgM (Fig 1). The strongest negative correlation is shown for IgA and IgG2, the antibody classes that are least able to fix complement and consequently the strongest candidates as competitive inhibitors of *Salmonella* killing.

**Fig 2:**
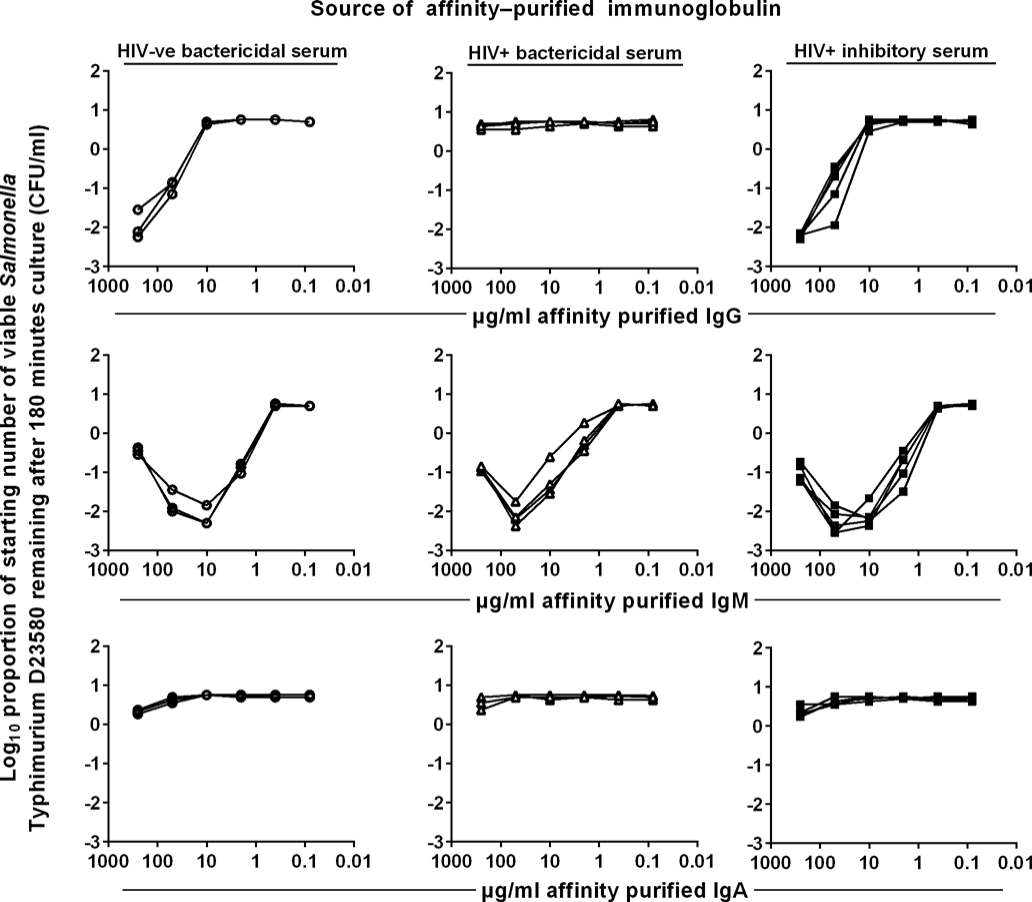
Killing of *S*. Typhimurium by total IgG, IgM and IgA from HIV-uninfected and HIV-infected sera. Log growth phase *S*. Typhimurium D23580 was added to a mixture of the purified antibodies and 75% BRS. The log_10_ proportion of the starting number of bacteria remaining after 180 minutes was determined on LB agar. To obtain total antibody of each isotype (IgG, IgM and IgA), serum was incubated sequentially with combinations of human IgA and IgM affinity matrices and protein G affinity matrix to remove IgA, IgM and IgG respectively. Sera used were HIV-uninfected (HIV-ve bactericidal, n = 3), HIV-infected bactericidal (HIV+ve bactericidal, n = 4) and HIV-infected inhibitory (HIV+ve inhibitory, n = 5) sera. Each line represents killing by purified total IgG, IgM or IgA antibodies from one serum. Data shown are from a single representative experiment, where each of the three independent experiments was performed in technical triplicates.

For more direct evidence about the capacity of different anti-LPS antibody classes to kill *S*. Typhimurium, total IgA, IgG and IgM were prepared from serum of three HIV-uninfected subjects (‘HIV-ve bactericidal’ serum), four HIV-infected subjects that effect normal killing of *S*. Typhimurium (‘HIV+ve bactericidal’ serum), and five HIV-infected subjects whose serum does not kill (‘HIV+ve inhibitory’ serum). Purified total IgG, IgM and IgA from each of these 12 sera were tested in a modified SBA with *S*. Typhimurium D23580 and dilutions of affinity purified immunoglobulin and 75% baby rabbit serum (BRS) as the source of complement [16] (Fig 2). Purified total IgG (top panel) from HIV-ve bactericidal sera and HIV+ve inhibitory sera were bactericidal against *S*. Typhimurium D23580 in the presence of BRS. Importantly, there is no loss of killing at 500μg/ml with all eight sera in these two groups, indicating that even high concentrations of these IgG preparations are not anti-complementary. Strikingly, none of the total IgG purified from HIV+ve bactericidal sera induced killing at any concentration tested. The highest IgG concentration tested (500 μg/ml) was below normal physiological levels in blood (6–16 mg/ml).

The killing observed in whole serum from many HIV-infected subjects may be primarily be due to IgM anti-LPS, since purified IgM HIV+ve bactericidal serum killed *S*. Typhimurium in the presence of BRS (Fig 2, central panel). Total IgM from HIV+ve inhibitory serum was also bactericidal, as was total IgM from HIV-ve bactericidal serum. All of these total IgM preparations induced somewhat less killing at the highest concentration tested (500 μg/ml) than at a five-fold lower concentration. None of the total IgA preparations from any of the three groups induced killing of *S*. Typhimurium D23580 at any concentration tested (Fig 2, bottom row). This is consistent with IgA not activating antibody-dependent classical complement pathway [18]. It also suggests that anti-LPS IgA in high concentration can act as a competitive inhibitor of antibody-induced complement-mediated killing of *S*. Typhimurium.

**Fig 3:**
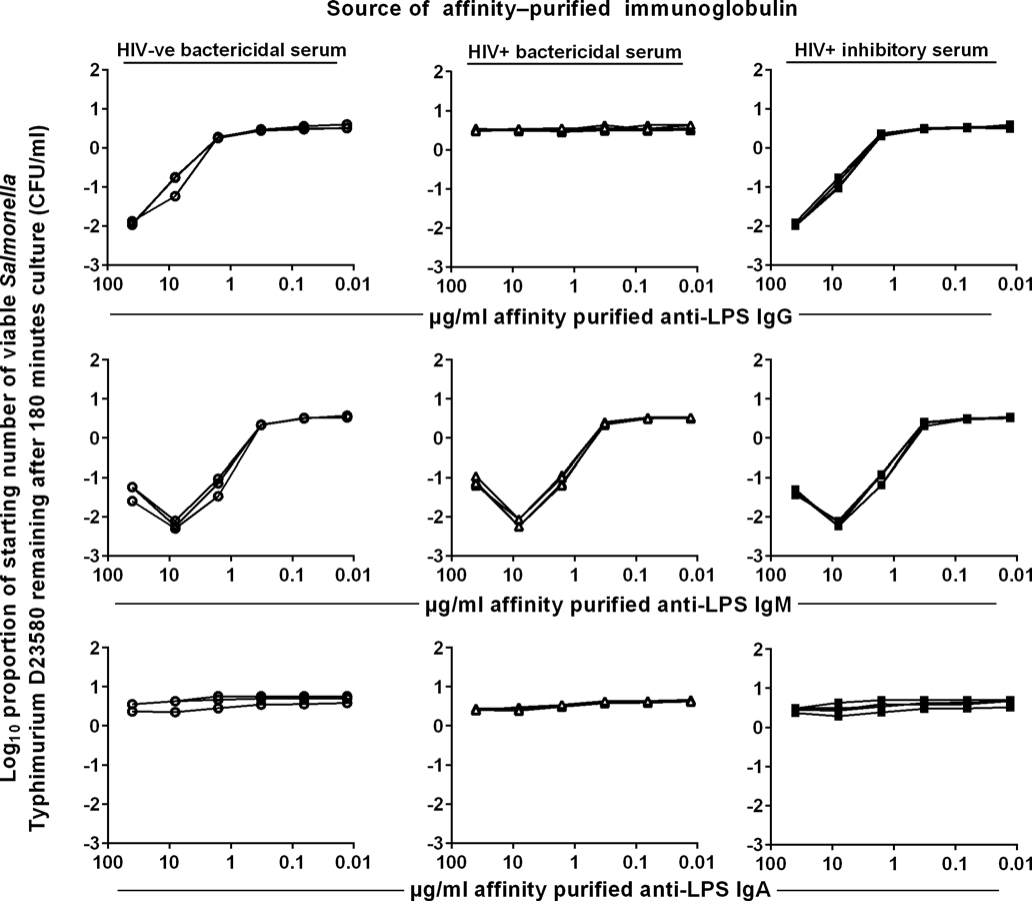
Killing of *S*. Typhimurium by anti-LPS isotypes from HIV-ve bactericidal, HIV+ve bactericidal and HIV+ve inhibitory sera. Log growth phase *S*. Typhimurium D23580 was added to a mixture of the purified antibodies and 75% BRS as complement source. The log_10_ proportion of the starting number of bacteria remaining after 180 minutes was determined on LB agar. Anti-*Salmonella* LPS antibodies were extracted from affinity-purified total IgG, IgA and IgM using a *S*. Typhimurium D23580 LPS O-antigen column. Each line represents killing by purified anti-LPS antibodies from one serum. Data shown are from a single representative experiment, where each of the three independent experiments was performed in technical triplicates.

**Fig 4:**
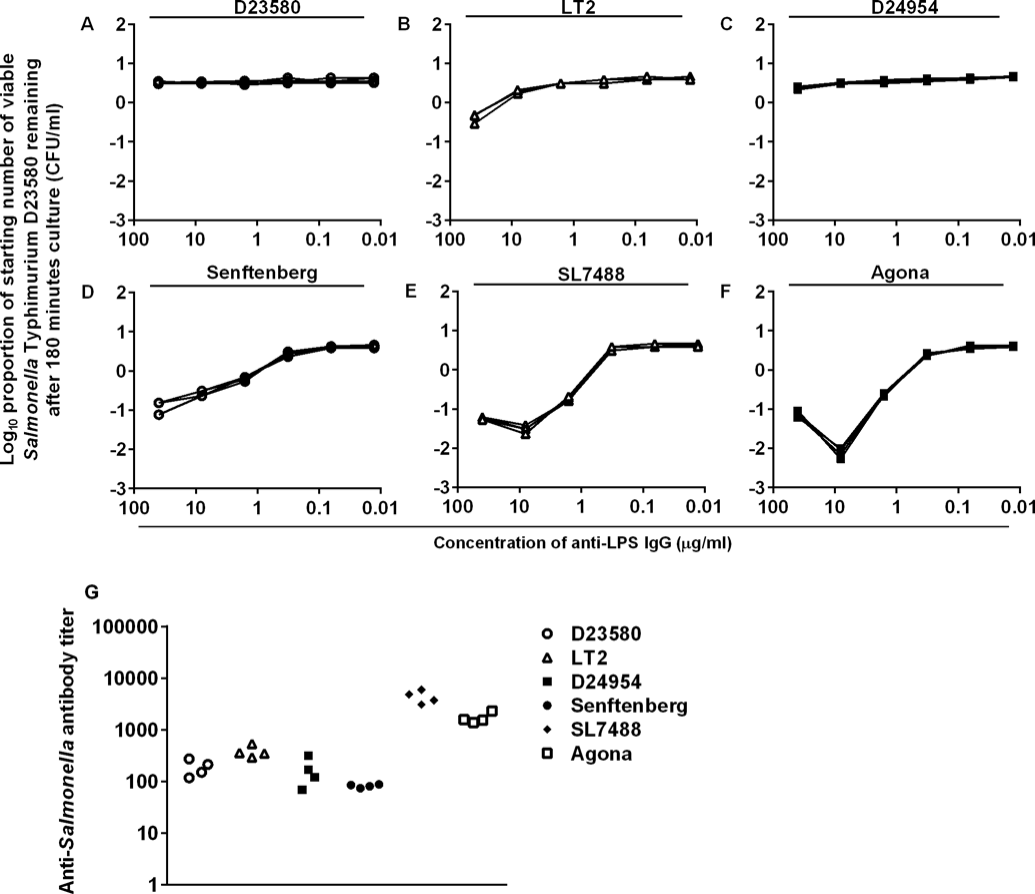
Killing of six strains of *Salmonella* by anti-LPS IgG from HIV+ve bactericidal sera. A) *S*. Typhimurium D23580, (B) *S*. Typhimurium LT2, (C) *S*. Enteritidis D24954, (D) *S*. Senftenberg 20050439, (E) *S*. Enteritidis SL7488, (F) *S*. Agona 20071186. Log growth phase *Salmonella* was added to a mixture of the purified antibodies and 75% BRS as complement source. The log_10_ proportion of the starting number of bacteria remaining after 180 minutes was determined on LB agar. (G) Flow cytometric determination of anti-LPS IgG, from HIV+ve bactericidal sera, to the *Salmonella* strains. Each line/point represents one serum (n = 4).

To relate these findings to *S*. Typhimurium LPS-specific antibodies, we affinity purified anti-LPS antibodies from each total immunoglobulin preparation and repeated the modified SBA. Remarkably, the results with anti-LPS antibodies of each class almost completely mirrored those with total immunoglobulins of each class, consistent with anti-LPS antibodies in each serum effecting the killing observed (Fig 3). The relative lack of bactericidal activity of anti-LPS IgG from HIV+ve bactericidal sera total IgG was again surprising. We speculated whether differences in the fine specificities of the *S*. Typhimurium O-antigen epitopes recognized by these IgG antibodies could be responsible. Since anti-LPS antibodies were affinity-purified using O-antigen from *S*. Typhimurium D23580 (consisting of O:1, O:4, O:5 and O:12 epitopes), anti-LPS IgG from HIV+ve bactericidal sera potentially could target a different balance of O-antigen epitopes compared with anti-LPS IgG from the other sera groups. To test this, we examined the ability of purified anti-LPS IgG from HIV+ve bactericidal sera to kill *Salmonella* with different O-antigen profiles (S2 Table, Fig 4A–4F).

As well as being unable to kill *S*. Typhimurium D23580, anti-LPS IgG from HIV+ve bactericidal sera exhibited weak bactericidal activity against *S*. Typhimurium LT2 (both O:1, O:4, O:5, O:12), possibly due to the higher serum sensitivity of LT2 compared with D23580 [16]. These IgG antibodies could not kill *S*. Enteritidis D24954 (O:1, O:9, O:12), but could kill *S*. Senftenberg (O:1, O:3, O:19). As O:1 antigen is the only O-antigen common between *S*. Senftenberg and *S*. Typhimurium D23580 (used to purify the anti-LPS antibodies), killing of *S*. Senftenberg was most likely mediated by antibodies to O:1 antigen, an α(1→6) glucosylated galactose found on the LPS backbone [9,19]. Absence of bactericidal activity with *S*. Typhimurium D23580 and *S*. Enteritidis D24954, which also express O:1, could be due to differences in O-antigen chain length, and glucosylation and O-acetylation levels which impact on the tridimensional structure of these side chains, reducing accessibility of IgG antibodies to the O:1 antigen. Anti-LPS IgG from HIV+ve bactericidal sera killed *S*. Enteritidis SL7488 (O:1, O:4, O:12) and *S*. Agona (O:4, O:12), likely due to antibodies to O:4 antigen which is present on both strains, but is less accessible to antibody in the presence of the O:5 antigen of *S*. Typhimurium D23580 and LT2. Analysis of the anti-LPS IgG from the HIV+ve bactericidal sera by flow cytometry (Fig 4G) showed higher binding to *S*. Enteritidis SL7488 and *S*. Agona compared to the other bacterial strains. This is consistent with antibody binding to the exposed O:4 epitope on these two strains.

### Inhibition of killing of *S*. Typhimurium by LPS-specific isotypes from sera of HIV-infected adults

**Fig 5:**
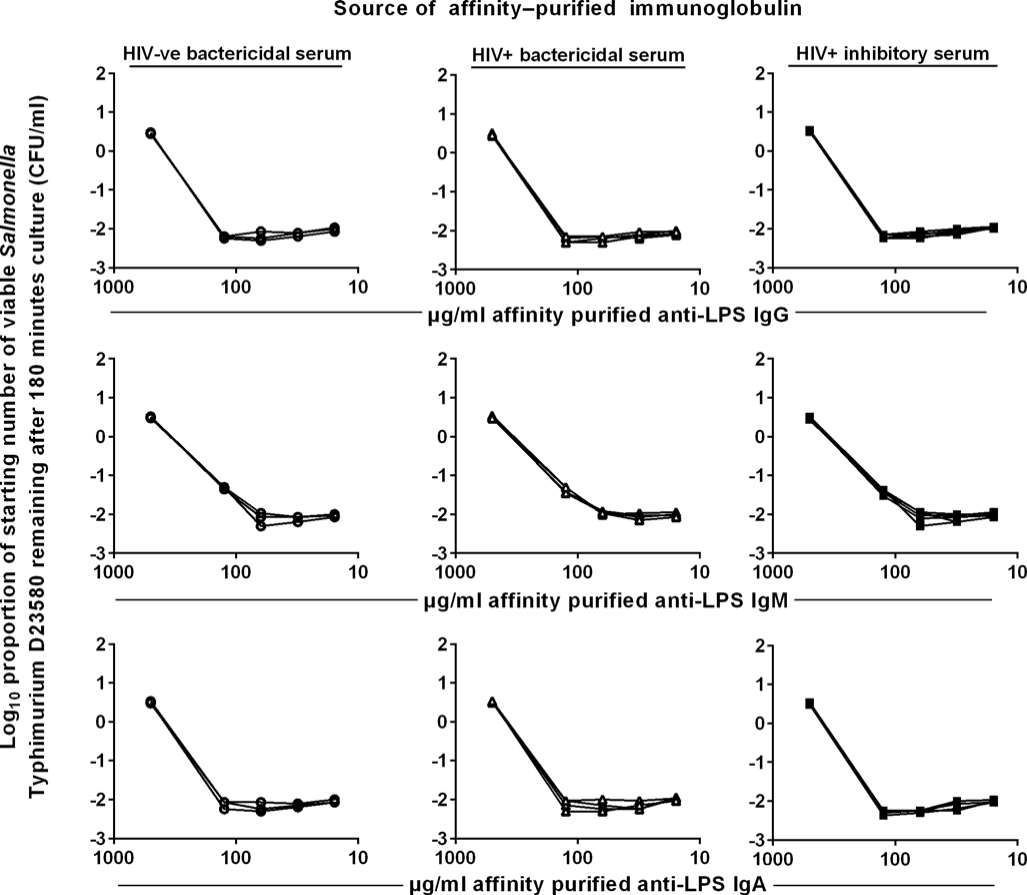
Inhibition of *S*. Typhimurium killing by anti-LPS antibodies from HIV-ve bactericidal, HIV+ve bactericidal and HIV+ve inhibitory sera. Log growth phase *S*. Typhimurium D23580 was added to a mixture of the purified anti-LPS antibodies (from HIV-ve bactericidal, HIV+ve bactericidal and HIV+ve inhibitory sera) and 50% HIV-uninfected human serum. The log_10_ proportion of the starting number of bacteria remaining after 180 minutes was determined on LB agar. Each line represents inhibition of killing by anti-LPS antibodies from one serum. Data shown are from a single representative experiment, where each of the three independent experiments was performed in technical triplicates.

To test the possibility that specific IgA acts as a competitive inhibitor of antibody-induced killing of *S*. Typhimurium D23580, we added purified anti-LPS antibodies from each of the total IgG, IgM and IgA preparations to SBA of *S*. Typhimurium D23580 with 50% HIV-ve bactericidal adult African serum as a source of both anti-*Salmonella* antibodies, that induce killing, and complement (Fig 5). Perhaps surprisingly, 500 μg/ml anti-LPS of each of the three immunoglobulin classes from the sera of all three groups inhibited antibody-induced complement-mediated killing of *S*. Typhimurium D23580. This inhibition was lost after a four-fold dilution of the anti-LPS IgA and IgG, and after one further dilution with anti-LPS IgM. As a control, purified non-*Salmonella* specific human antibody preparations were added to the SBA and failed to inhibit serum killing at 500 μg/ml.

### Complement integrity in sera with high anti-LPS concentrations that inhibit *S*. Typhimurium killing

The finding that high concentrations of anti-LPS antibody of all isotypes from each clinical group inhibits complement-induced killing, prompted us to explore three possible mechanisms for this effect. First, we tested whether HIV+ve inhibitory sera consume complement. Second, we investigated whether concentrations of anti-LPS antibodies that saturate O-antigen epitopes on *S*. Typhimurium prevent formation of membrane attack complex (MAC) of complement. Third, we checked whether MAC is formed, but prevented from inserting in the bacterial cell wall in a damaging way by saturating concentrations of anti-LPS antibody.

**Fig 6:**
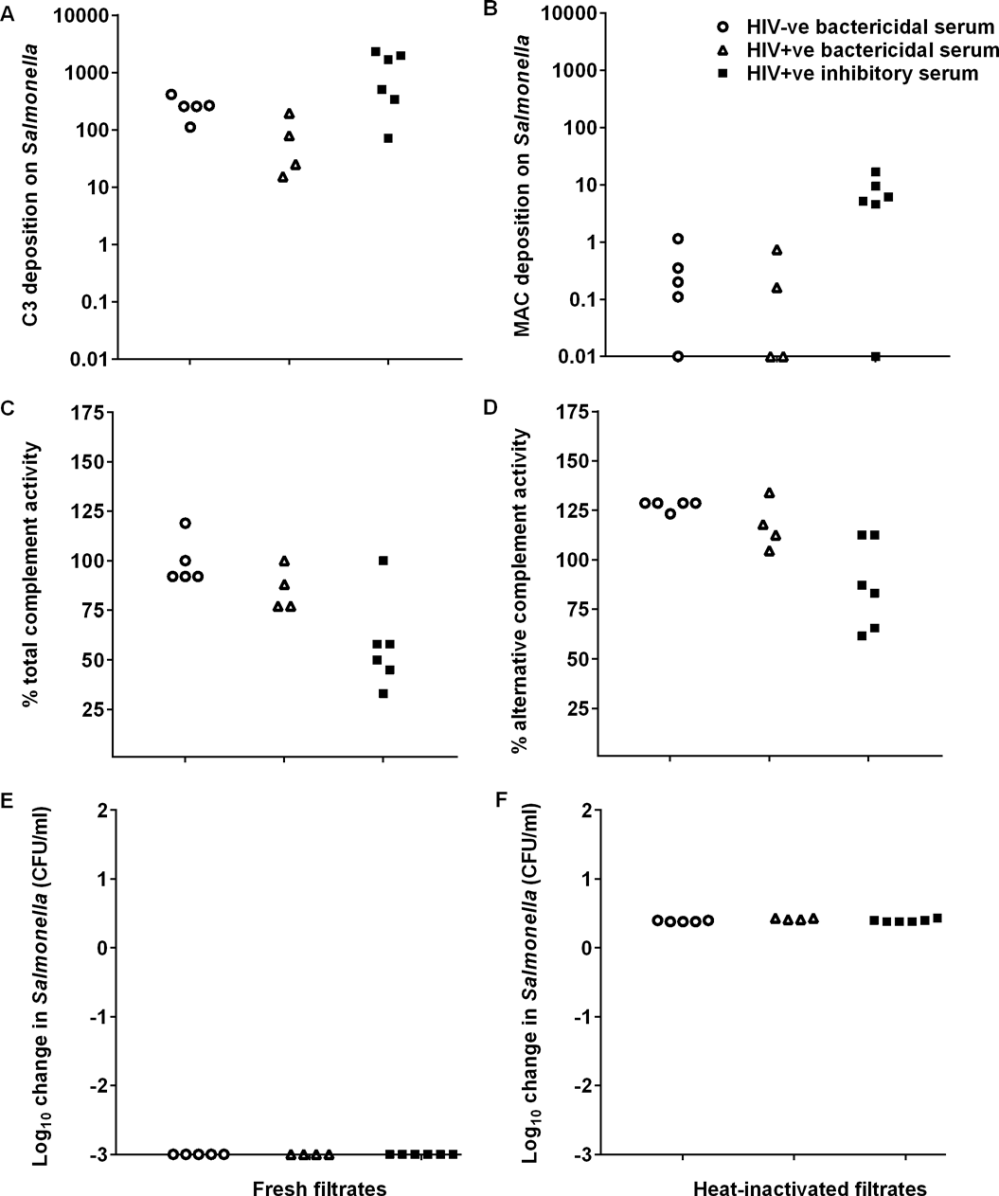
Complement integrity of HIV-ve bactericidal, HIV+ve bactericidal and HIV+ve inhibitory sera following SBA with *S*. Typhimurium. Log growth phase *S*. Typhimurium D23580 was added to whole serum and incubated for 180 min in a SBA. Following the SBA, sera were filter-sterilized and examined for complement integrity. (A) C3 and (B) MAC deposition on D23580 detected by flow cytometry using anti-C3 and anti-MAC (anti-C5b-9) antibodies followed by FITC-conjugated anti-mouse immunoglobulin. (C) Total hemolytic and (D) alternative complement activity measured by radial immunodiffusion assay. Bactericidal potential at 45 min in a new whole serum SBA with *galE*^-^ D23580 using (E) fresh and (F) heat-inactivated filtrates. Data shown are from a single representative experiment, where each of the three independent experiments was performed in technical triplicates.

To test for complement depletion in sera, SBA against *S*. Typhimurium D23580 were first set up with the different sera. After 180 mins, the sera were sterile-filtered and complement function assessed. All post-SBA filtrates could deposit C3 complement on *S*. Typhimurium as assessed by flow cytometry. Most of the filtrates, including 5 of 6 from the HIV+ve inhibitory group, also deposited MAC. There was no relative impairment of complement deposition with HIV+ve inhibitory filtrates compared with those of the other two groups (Fig 6A and 6B). All post-SBA filtrates retained total and alternative pathway hemolytic complement activity (Fig 6C and 6D), although there was a trend for lower activity in the HIV+ve inhibitory filtrates compared with the HIV-ve bactericidal filtrates. We previously demonstrated that 20% human complement can effect bactericidal activity against *S*. Typhimurium D23580 in the presence of specific antibodies [5]. Finally, post-SBA filtrates were tested for killing capacity in a second SBA with *S*. Typhimurium D23580 *galE*^*-*^. This strain is sensitive to complement-mediated killing in the absence of antibodies [11]. All filtrates in each group, including the HIV+ve inhibitory group, effected maximal killing of *S*. Typhimurium D23580 *galE*^*-*^ after 45 minutes. Heat-inactivation at 56°C for 30 minutes destroyed the lytic capacity of the filtrates (Fig 6E and 6F).

### Anti-LPS-associated impaired *Salmonella* killing is not due to low antibody affinity or avidity

**Fig 7:**
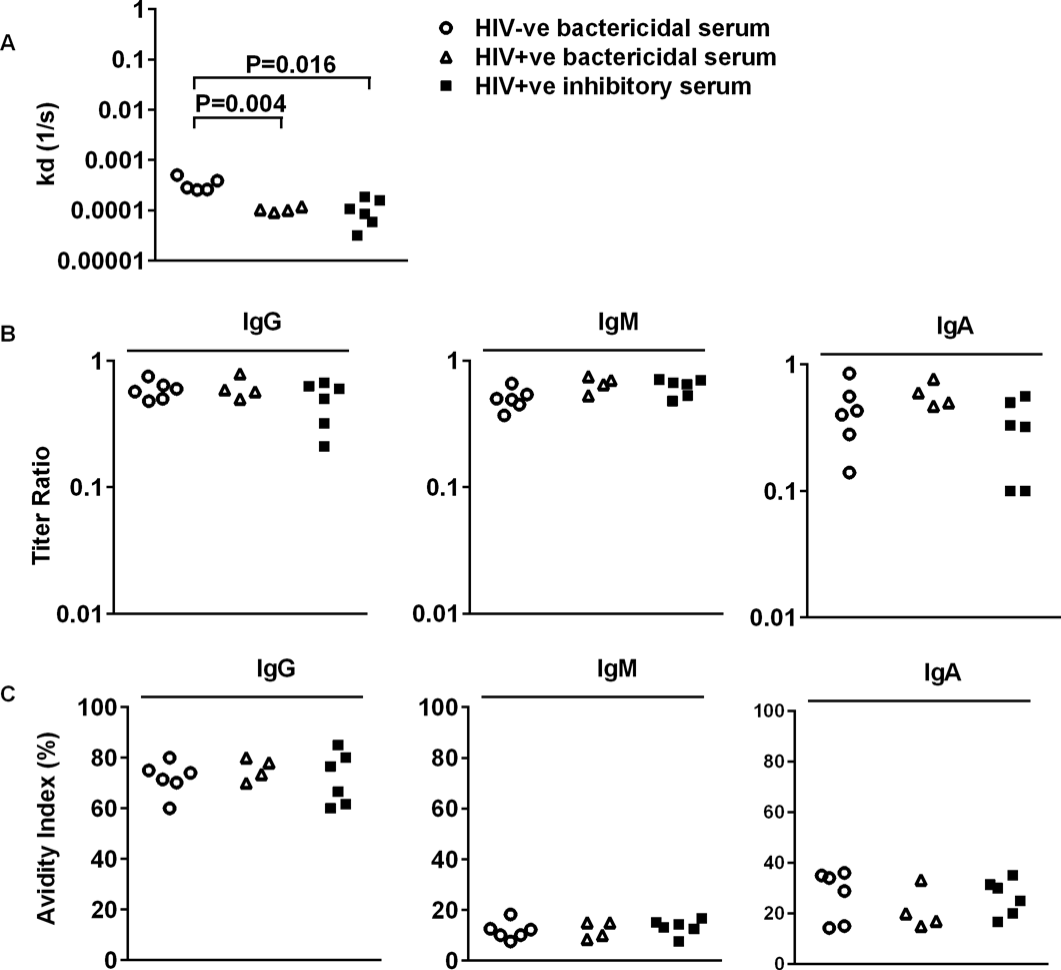
k_d_, affinity and avidity of anti-LPS antibodies from HIV-infected and HIV-uninfected adults. (A) *k*_*d*_ values for total anti-LPS antibodies in HIV-ve bactericidal, HIV+ve bactericidal, HIV+ve inhibitory sera. Binding of serum antibodies to *S*. Typhimurium LPS were assessed using a Biacore 3000 system. A hydrophobic HPA sensor chip was coated by passing LPS across the chip surface, washed with hydrochloride acid and blocked with bovine serum albumin. Sera were diluted 1:2 and passed across the chip surface. *k*_*d*_ values were calculated by fitting the binding curves to a best-fit Langmuir 1:1 model using BiaEvaluation. (B) Affinity and (C) avidity of isotype-specific anti-LPS antibodies, as measured by ELISA. IgG4 was not detected. For affinity measurement, ELISA plates were coated with *S*. Typhimurium LPS at either a non-limiting concentration of 5 μg/ml or limiting concentration of 0.5 μg/ml. Affinity was calculated as the ratio of the antibody titer with limiting plates to titer with non-limiting plates. For avidity measurement, ELISA plates were coated with *S*. Typhimurium LPS at 5 μg/ml and ELISA was performed using diluted human serum. For this ELISA, half the wells were washed with 6 M urea and half with PBS-0.05% Tween 20. The avidity index is antibody titer in the presence of urea as a percentage of titer in the absence of urea. Comparison between groups by Mann-Whitney U-test.

**Fig 8:**
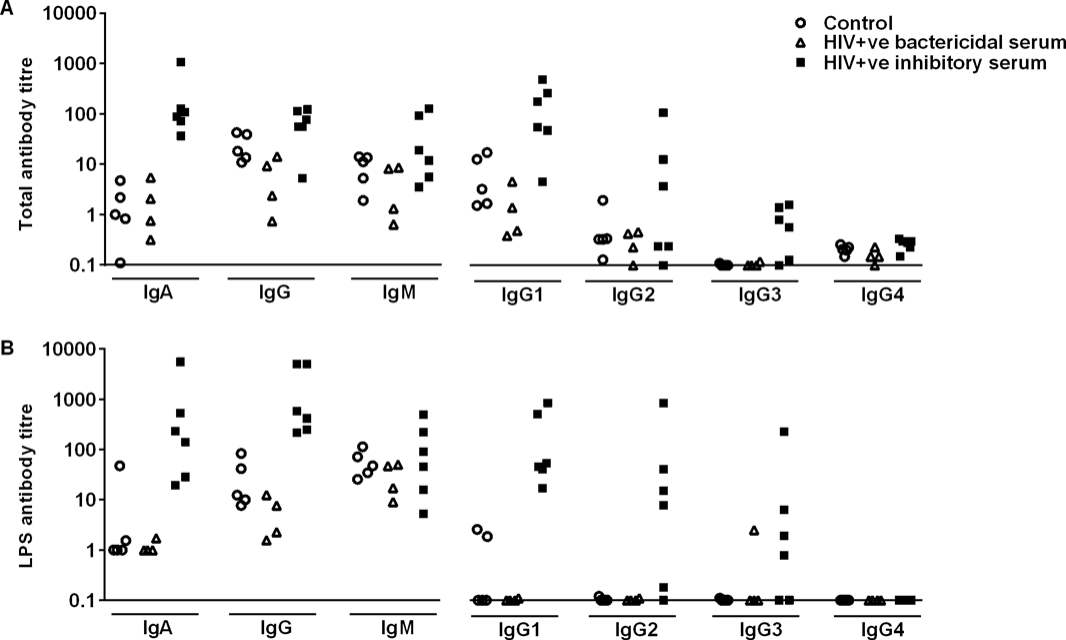
Total anti-*S*. Typhimurium and specific anti-*S*. Typhimurium LPS antibody in sera from HIV-infected and HIV-uninfected adults. (A) Total anti-*S*. Typhimurium antibody levels in the different groups of sera (HIV-ve bactericidal, HIV+ve bactericidal and HIV+ve inhibitory) by flow cytometry. (B) Specific anti-*S*. Typhimurium LPS antibody levels by ELISA. Anti-LPS antibody concentration of the sera were determined using anti-LPS ELISA, where *S*. Typhimurium LPS was coated onto ELISA plates at 5 μg/ml. Anti-LPS antibodies were detected using alkaline-phosphatase-conjugated anti-human isotype-specific antibodies. Each point represents one serum.

We speculated that differences in affinity and avidity of antibodies targeting *S*. Typhimurium LPS might contribute to the lack of serum bactericidal activity. Using Biacore, we determined the dissociation constant (*k*_*d*_*)* of anti-*S*. Typhimurium D23580 LPS antibodies (Fig 7A) in each group of sera (antibody titers shown in Fig 8 and S3 Table). The *k*_*d*_ of anti-LPS antibodies in HIV+ve inhibitory (P = 0.004) and HIV+ve bactericidal sera (P = 0.016) were lower than for HIV-ve bactericidal sera. However there were no differences, as measured by ELISA, in affinity (Fig 7B) and avidity (Fig 7C) of anti-LPS IgA, IgG and IgM between the three groups.

## Discussion

*S*. Typhimurium LPS O-antigen has been considered as a vaccine candidate for many years. We previously reported an association between impaired serum killing of *S*. Typhimurium and high levels of *S*. Typhimurium LPS-specific IgG in some African HIV-infected adults [11]. This study extends the association to high titers of anti-LPS IgA, but not IgM. Factors we examined that do not appear to cause impaired killing are antibody affinity, avidity and complement consumption. The latter was previously postulated as an explanation [11].

The first key finding is that antibody concentration is an important determinant of the presence or absence of serum killing of *Salmonella*. HIV+ve inhibitory sera have elevated LPS-specific IgA and IgG, compared with HIV-ve and HIV+ve bactericidal sera. While whole undiluted HIV+ve inhibitory sera cannot kill *S*. Typhimurium D23580, these sera kill *Salmonella* in the presence of exogenous complement when diluted. The concentration dependency of killing is also observed with purified antibodies from all groups of sera. Killing is also isotype-dependent, being mediated by IgG and IgM in HIV-ve bactericidal sera, but predominantly by IgM in the HIV+ve bactericidal sera tested.

The counter-intuitive lack of killing observed with purified total and LPS-specific IgG from HIV+ve bactericidal sera could be due to the epitopes recognized by these antibodies, suggesting that the specific O-antigen epitopes recognized are important for efficient killing of *Salmonella*. The SBA and flow cytometry experiments with *Salmonella* strains of different O-antigen profiles supports this hypothesis and offers a possible explanation whereby preferential targeting of anti-LPS IgG from the HIV+ve bactericidal group to O:4 could underlie this finding. The O:5 antigen, present in *S*. Typhimurium D23580, is an acetylated form of the O:4 antigen [6, 20], and can hinder accessibility of antibodies targeting O:4. The underlying reasons for this difference in epitope specificity of IgG anti-LPS antigens in HIV+ve bactericidal and HIV+ve inhibitory sera are not clear. The difference may be partly explained by the small number of sera available for antibody extraction. With larger numbers of sera, it is conceivable that both bactericidal and inhibitory sera would be found with antibodies preferentially targeting the O:4 or O:5 epitopes. The presence of a skewing in antibody response to particularly epitopes is consistent with the well-recognized B cell dysregulation and dysfunction observed in HIV infection [21, 22], characterized by various abnormalities including oligoclonal antibody responses [23].

The second key finding is that anti-LPS antibodies from HIV-infected and HIV-uninfected African adults can effect complement-dependent killing, indicating their potential for mediating protective immunity against NTS. Purified anti-LPS IgG and IgM kill *Salmonella*, while IgA does not. Hence, bactericidal activity is dependent on antibody isotype, which is consistent with the known ability of IgM, IgG1 and IgG3, but not IgA, and only to a limited extent IgG2, to activate complement [24–26]. While purified IgA from African adults could not effect complement-mediated killing of *Salmonella*, we recently found that a vaccine-induced mouse monoclonal IgA against O:4 of *S*. Typhimurium LPS has bactericidal activity [8]. This could simply be due to species differences between human and mouse IgA. While serum IgA did not kill *Salmonella*, secretory IgA, which was not examined in this study, is likely to be important for reducing *Salmonella* colonization via the oral route.

Concentration of anti-LPS IgA and IgG2 in total serum from HIV-infected participants correlated most strongly with inhibition of *Salmonella* killing (P<0.0001 for both) and it is possible that the high levels of this immunoglobulin class and subclass in HIV+ve inhibitory serum contributes to impaired killing. Very few HIV-uninfected participants had raised IgG2 levels in comparison with HIV-infected participants. Although IgG1 is the most abundant IgG subclass in human serum, IgG2 appears to be induced preferentially to bacterial polysaccharides [27]. As well as its poor ability to activate complement, high levels of IgG2 to *Pseudomonas aeruginosa* LPS in patients with bronchiectasis have been found recently to inhibit serum killing of these bacteria [28].

The relevance of different antibody isotypes for protection has implications for vaccine development against invasive *Salmonella* disease. Vaccine design, formulation and regimes that induce antibody responses consisting predominantly of IgG1 and IgM are more likely to elicit bactericidal antibodies which can counter bacteremia, while IgA induction is likely to be important for preventing the initial invasion from the gastrointestinal tract.

The third key finding is that while anti-LPS IgG and IgM are bactericidal at low concentrations, high concentrations inhibit normal serum killing. Together, these findings suggest that impaired killing with HIV+ve inhibitory sera is primarily the outcome of high anti-LPS antibodies concentrations. The exact mechanism by which this inhibition occurs is still unclear, but could partly be due to the very high levels of anti-LPS IgA and IgG2 antibodies in HIV+ve inhibitory sera which are non/poorly-complement activating. This does not, however, appear to explain the similar profiles of killing and lack of killing by anti-LPS antibodies extracted from HIV-infected and HIV-uninfected sera over the range of concentrations tested, with the possible exception of lack of killing of IgG with HIV+ve bactericidal sera if this was predominantly IgG2 subclass.

As previously postulated [11], binding of an excess of antibodies to the *Salmonella* surface could sterically hinder insertion of the MAC into the bacterial membrane, so that although MAC is formed, as we have detected, it is unable to kill *Salmonella*. Lack of killing could also result from deposition of MAC at a distance away from the bacterial surface, since anti-LPS antibodies will bind theoretically along the length of the LPS molecule, with some locations distal to the bacterial surface. Location of MAC deposition has previously been highlighted as an important factor in killing of *Salmonella* Typhimurium [29]. However, this alone cannot be the full explanation, since anti-LPS antibodies are bactericidal [7, 8, 30], unless distal MAC deposition occurs preferentially at higher anti-LPS antibody concentrations. Such distally-positioned MAC may be prone to shedding from the bacteria as previously demonstrated for *S*. Minnesota [31, 32]. Finally, it is conceivable that high concentrations of antibodies bound to individual bacteria may result in defective formation of the MAC, leading to a lack of killing.

Trebicka et al. have recently described the absence of killing of *S*. Typhimurium by sera from HIV-infected Americans associated with a lack of IgG antibodies to LPS [33], rather than the high levels of these antibodies that we have found in HIV-infected Africans. This is consistent with the concept that some, but not excessive, antibodies to LPS are required to kill *Salmonella*. The reason for the absence of antibodies to LPS may relate to differences in exposure to *Salmonella* in the USA compared with Africa, leading to reduced antigenic stimulation of LPS-specific B cells, or to differences in the nature of B cell dysfunction and dysregulation between the two study populations. Interestingly, despite the lack of anti-LPS antibodies, Trebicka et al found that these HIV-infected sera inhibited killing of *Salmonella* by control sera, suggesting multiple mechanisms for impairment of killing of *Salmonella* in HIV infection. Although data on levels of IgA and IgM antibodies to LPS were not shown, the authors speculated that high levels anti-LPS IgM present in some study participants could be the reason for inhibition of killing. This is consistent with the findings in our current study.

The LPS-specific hypergammaglobulinemia found in some HIV-infected African adults is not present in HIV-uninfected subjects, and appears to occur secondary to natural exposure to *S*. Typhimurium in the context of the dysregulated humoral immunity that accompanies HIV infection [11]. Inhibition of normal serum killing of *Salmonella* by anti-LPS IgG and IgM required 500 μg/ml and 125 μg/ml purified antibodies respectively. The combined concentrations of all anti-LPS antibody isotypes exceeded 500 μg/ml in HIV+ve inhibitory sera, but were below 500 μg/ml in HIV+ve and HIV-ve bactericidal sera. Immunization has been reported to induce ∼15 μg/ml specific IgG in healthy adults [34, 35]. Therefore, a *S*. Typhimurium O-antigen-based vaccine should induce antibodies at bactericidal rather than inhibitory concentrations in HIV-uninfected individuals.

Several factors could determine the immunological response to such a vaccine in HIV-infected individuals. HIV infection has a global effect on the immune system characterized by impaired immune homeostasis, and is associated with higher risk and increased severity of infections including pneumonia and tuberculosis [36, 37]. The difference in immunity resulting from vaccination instead of natural exposure to NTS is uncertain, as is the effect antiretroviral therapy will have on anti-*Salmonella* LPS antibody levels. Therefore, it is not known whether vaccination will induce a protective response or a dysregulated excess of anti-LPS antibodies that impairs serum *Salmonella* killing.

In conclusion, antibodies against *S*. Typhimurium LPS O-antigen are present in the blood of HIV-infected and HIV-uninfected African adults, most likely following natural exposure to *S*. Typhimurium. The IgG and IgM isotypes of these antibodies have in vitro bactericidal activity against invasive African *S*. Typhimurium, but at high concentrations, all three isotypes (IgG, IgA and IgM) can inhibit killing of *Salmonella*.

## Supporting Information

S1 Table Subject details. (DOCX)

S2 Table Expression of O antigens by *Salmonella* strains. (DOCX)

S3 Table Anti-*S*. Typhimurium LPS antibody concentrations in undiluted sera and in purified antibody isotype fractions. (DOCX)

## References

[1] Feasey NA, Dougan G, Kingsley RA, Heyderman RS, Gordon MA. Invasive non-typhoidal salmonella disease: an emerging and neglected tropical disease in Africa. Lancet. 2012;379: 2489–2499. 10.1016/S0140-6736(11)61752-2 22587967 PMC3402672

[2] MacLennan CA, Levine MM. Invasive nontyphoidal Salmonella disease in Africa: current status. Expert Rev Anti Infect Ther. 2013;11: 443–446. 10.1586/eri.13.27 23627848

[3] Gordon MA, Banda HT, Gondwe M, Gordon SB, Boeree MJ, Walsh AL, et al Non-typhoidal salmonella bacteraemia among HIV-infected Malawian adults: high mortality and frequent recrudescence. AIDS. 2002;16: 1633–1641. 12172085 10.1097/00002030-200208160-00009

[4] Kariuki S, Gordon MA, Feasey N, Parry CM. Antimicrobial resistance and management of invasive Salmonella disease. Vaccine. 2015;33 Suppl 3: C21–29. 10.1016/j.vaccine.2015.03.102 25912288 PMC4469558

[5] MacLennan CA, Gondwe EN, Msefula CL, Kingsley RA, Thomson NR, White SA, et al The neglected role of antibody in protection against bacteremia caused by nontyphoidal strains of Salmonella in African children. J Clin Invest. 2008;118: 1553–1562. 10.1172/JCI33998 18357343 PMC2268878

[6] Watson DC, Robbins JB, Szu SC. Protection of mice against Salmonella typhimurium with an O-specific polysaccharide-protein conjugate vaccine. Infect Immun. 1992;60: 4679–4686. 1383154 10.1128/iai.60.11.4679-4686.1992 PMC258218

[7] Rondini S, Micoli F, Lanzilao L, Gavini M, Alfini R, Brandt C, et al Design of glycoconjugate vaccines against invasive African Salmonella enterica serovar Typhimurium. Infect Immun. 2015;83: 996–1007. 10.1128/IAI.03079-14 25547792 PMC4333450

[8] Goh YS, Clare S, Micoli F, Saul A, Mastroeni P, MacLennan CA. Monoclonal Antibodies of a Diverse Isotype Induced by an O-Antigen Glycoconjugate Vaccine Mediate In Vitro and In Vivo Killing of African Invasive Nontyphoidal Salmonella. Infect Immun. 2015;83: 3722–3731. 10.1128/IAI.00547-15 26169269 PMC4534659

[9] Carlin NI, Svenson SB, Lindberg AA. Role of monoclonal O-antigen antibody epitope specificity and isotype in protection against experimental mouse typhoid. Microb Pathog. 1987;2: 171–183. 2467161 10.1016/0882-4010(87)90019-2

[10] Nyirenda TS, Gilchrist JJ, Feasey NA, Glennie SJ, Bar-Zeev N, Gordon MA, et al Sequential acquisition of T cells and antibodies to nontyphoidal Salmonella in Malawian children. J Infect Dis. 2014;210: 56–64. 24443544 10.1093/infdis/jiu045 PMC4054899

[11] MacLennan CA, Gilchrist JJ, Gordon MA, Cunningham AF, Cobbold M, Goodall M, et al Dysregulated humoral immunity to nontyphoidal Salmonella in HIV-infected African adults. Science. 2010;328: 508–512. 10.1126/science.1180346 20413503 PMC3772309

[12] Kingsley RA, Msefula CL, Thomson NR, Kariuki S, Holt KE, Gordon MA, et al Epidemic multiple drug resistant Salmonella Typhimurium causing invasive disease in sub-Saharan Africa have a distinct genotype. Genome Res. 2009;19: 2279–2287. 10.1101/gr.091017.109 19901036 PMC2792184

[13] Hoiseth SK, Stocker BA. Aromatic-dependent Salmonella typhimurium are non-virulent and effective as live vaccines. Nature. 1981;291: 238–239. 7015147 10.1038/291238a0

[14] Hormaeche CE, Mastroeni P, Harrison JA, Demarco de Hormaeche R, Svenson S, Stocker BA. Protection against oral challenge three months after i.v. immunization of BALB/c mice with live Aro Salmonella typhimurium and Salmonella enteritidis vaccines is serotype (species)-dependent and only partially determined by the main LPS O antigen. Vaccine. 1996;14: 251–259. 8744548 10.1016/0264-410x(95)00249-z

[15] Berger CN, Brown DJ, Shaw RK, Minuzzi F, Feys B, Frankel G. Salmonella enterica strains belonging to O serogroup 1,3,19 induce chlorosis and wilting of Arabidopsis thaliana leaves. Environ Microbiol. 2011;13: 1299–1308. 10.1111/j.1462-2920.2011.02429.x 21349136

[16] Goh YS, MacLennan CA. Invasive African nontyphoidal Salmonella requires high levels of complement for cell-free antibody-dependent killing. J Immunol Methods. 2013;387: 121–129. 10.1016/j.jim.2012.10.005 23085530

[17] O'Shaughnessy CM, Micoli F, Gavini M, Goodall M, Cobbold M, Saul A, et al Purification of antibodies to O antigen of Salmonella Typhimurium from human serum by affinity chromatography. J Immunol Methods. 2013;387: 199–210. 10.1016/j.jim.2012.10.015 23142459

[18] Cunningham AF, Gaspal F, Serre K, Mohr E, Henderson IR, Scott-Tucker A, et al Salmonella induces a switched antibody response without germinal centers that impedes the extracellular spread of infection. J Immunol. 2007;178: 6200–6207. 17475847 10.4049/jimmunol.178.10.6200

[19] Hansen KR, Nielsen LR, Lind P. Use of IgG avidity ELISA to differentiate acute from persistent infection with Salmonella Dublin in cattle. J Appl Microbiol. 2006;100: 144–152. 16405694 10.1111/j.1365-2672.2005.02758.x

[20] Hellerqvist CG, Lindberg B, Svensson S, Holme T, Lindberg AA. Structural studies on the O-specific side chains of the cell wall lipopolysaccharides from Salmonella typhi and S. enteritidis. Acta Chem Scand. 1969;23: 1588–1596. 5360616 10.3891/acta.chem.scand.23-1588

[21] Moir S, Fauci AS. B cells in HIV infection and disease. Nat Rev Immunol. 2009;9: 235–245. 10.1038/nri2524 19319142 PMC2779527

[22] Doria-Rose NA, Connors M. Antibody-secreting B cells in HIV infection. Curr Opin HIV AIDS. 2009;4: 426–430. 10.1097/COH.0b013e32832d9fac 20048707 PMC2885891

[23] Kouns DM, Marty AM, Sharpe RW. Oligoclonal bands in serum protein electrophoretograms of individuals with human immunodeficiency virus antibodies. JAMA. 1986;256: 2343. 3464766

[24] Lucisano Valim YM, Lachmann PJ. The effect of antibody isotype and antigenic epitope density on the complement-fixing activity of immune complexes: a systematic study using chimaeric anti-NIP antibodies with human Fc regions. Clin Exp Immunol. 1991;84: 1–8. 1707767 10.1111/j.1365-2249.1991.tb08115.x PMC1535367

[25] Bruggemann M, Williams GT, Bindon CI, Clark MR, Walker MR, Jefferis R, et al Comparison of the effector functions of human immunoglobulins using a matched set of chimeric antibodies. J Exp Med. 1987;166: 1351–1361. 3500259 10.1084/jem.166.5.1351 PMC2189658

[26] Schroeder HW Jr., Cavacini L. Structure and function of immunoglobulins. J Allergy Clin Immunol. 2010;125: S41–52. 10.1016/j.jaci.2009.09.046 20176268 PMC3670108

[27] Siber GR, Schur PH, Aisenberg AC, Weitzman SA, Schiffman G. Correlation between serum IgG-2 concentrations and the antibody response to bacterial polysaccharide antigens. N Engl J Med. 1980;303: 178–182. 6966763 10.1056/NEJM198007243030402

[28] Wells TJ, Whitters D, Sevastsyanovich YR, Heath JN, Pravin J, Goodall M, et al Increased severity of respiratory infections associated with elevated anti-LPS IgG2 which inhibits serum bactericidal killing. J Exp Med. 2014;211: 1893–1904. 10.1084/jem.20132444 25113975 PMC4144740

[29] Joiner KA, Tartanian AB, Hammer CH, Schweinle JE. Multimeric C9 within C5b-9 deposits in unique locations in the cell wall of Salmonella typhimurium. J Immunol. 1989;142: 4450–4457. 2656866

[30] Rondini S, Lanzilao L, Necchi F, O'Shaughnessy CM, Micoli F, Saul A, et al Invasive African Salmonella Typhimurium induces bactericidal antibodies against O-antigens. Microb Pathog. 2013;63: 19–23. 10.1016/j.micpath.2013.05.014 23756206

[31] Joiner KA, Hammer CH, Brown EJ, Frank MM. Studies on the mechanism of bacterial resistance to complement-mediated killing. II. C8 and C9 release C5b67 from the surface of Salmonella minnesota S218 because the terminal complex does not insert into the bacterial outer membrane. J Exp Med. 1982;155: 809–819. 6801180 10.1084/jem.155.3.809 PMC2186624

[32] Joiner KA, Hammer CH, Brown EJ, Cole RJ, Frank MM. Studies on the mechanism of bacterial resistance to complement-mediated killing. I. Terminal complement components are deposited and released from Salmonella minnesota S218 without causing bacterial death. J Exp Med. 1982;155: 797–808. 6801179 10.1084/jem.155.3.797 PMC2186629

[33] Trebicka E, Shanmugam NK, Mikhailova A, Alter G, Cherayil BJ. Effect of human immunodeficiency virus infection on plasma bactericidal activity against Salmonella enterica serovar Typhimurium. Clin Vaccine Immunol. 2014;21: 1437–1442. 10.1128/CVI.00501-14 25121777 PMC4266356

[34] Naess LM, Aarvak T, Aase A, Oftung F, Hoiby EA, Sandin R, et al Human IgG subclass responses in relation to serum bactericidal and opsonic activities after immunization with three doses of the Norwegian serogroup B meningococcal outer membrane vesicle vaccine. Vaccine. 1999;17: 754–764. 10067680 10.1016/s0264-410x(98)00259-x

[35] Shahid NS, Steinhoff MC, Hoque SS, Begum T, Thompson C, Siber GR. Serum, breast milk, and infant antibody after maternal immunisation with pneumococcal vaccine. Lancet. 1995;346: 1252–1257. 7475716 10.1016/s0140-6736(95)91861-2

[36] Flateau C, Le Loup G, Pialoux G. Consequences of HIV infection on malaria and therapeutic implications: a systematic review. Lancet Infect Dis. 2011;11: 541–556. 10.1016/S1473-3099(11)70031-7 21700241

[37] Gray DM, Zar HJ. Community-acquired pneumonia in HIV-infected children: a global perspective. Curr Opin Pulm Med. 2010;16: 208–216. 10.1097/MCP.0b013e3283387984 20375782

