# Supplementary material for: Bactericidal Immunity to Salmonella in Africans and Mechanisms Causing Its Failure in HIV Infection
Source: PLoS Negl Trop Dis. 2016 Apr 8;10(4):e0004604. doi: 10.1371/journal.pntd.0004604 (PMC4825999; doi:10.1371/journal.pntd.0004604)
Supplement: S1 Table — (DOCX) [file pntd.0004604.s001.docx]

**S1 Table**. **Subject details**

|  | **HIV-uninfected** | **HIV-infected** | |  |
| --- | --- | --- | --- | --- |
|  |  | **Impaired *Salmonella* killing** | **Normal *Salmonella* killing** | **All** |
| **Number** | 58 | 16 | 42 | 58 |
| **Median Age (range)** | 27 (17-53) | 34 (27-58) | 33 (17-60) | 33 (17-60) |
| **Gender  (male/female)** | 36/22 | 10/6 | 15/27 | 25/33 |
| **CD4 count cells/µl (range)** | 772 (329-1536) | 130 (1-817) | 237 (25-930) | 221 (1-930) |
| **Number with CD4 count <200 cell/ml** | 0 | 11 | 13 | 24 |
| **History of *Salmonella* disease** | 0 | 0 | 0 | 0 |

HIV-infected subjects were consenting patients at the antiretroviral clinic at Queen Elizabeth Central Hospital (QECH) who fulfilled the criteria for taking part in the study. Some has been diagnosed with HIV infection while in-patients at QECH, but all had recovered from their acute illness at the time of study recruitment. HIV-uninfected subjects were hospital staff and healthy relatives of patients attending the hospital. The exclusion criteria were fever (≥38 °C), malaria parasitemia, pregnancy, acute illness and current medication including antiretroviral therapy and cotrimoxazole (except one patient on isoniazid and ethambutol continuation phase tuberculosis treatment and one patient on fluconazole continuation phase cryptococcal treatment.
