# Supplementary material for: Bactericidal Immunity to Salmonella in Africans and Mechanisms Causing Its Failure in HIV Infection
Source: PLoS Negl Trop Dis. 2016 Apr 8;10(4):e0004604. doi: 10.1371/journal.pntd.0004604 (PMC4825999; doi:10.1371/journal.pntd.0004604)
Supplement: S2 Table — (DOCX) [file pntd.0004604.s002.docx]

**S2 Table. Expression of O antigens by *Salmonella* strains**

|  | **O:1** | **O:4** | **O:5** | **O:9** | **O:12** |
| --- | --- | --- | --- | --- | --- |
| ***S.* Typhimurium D23580** | **+** | **+** | **+** | **-** | **+** |
| ***S.* Typhimurium LT2** | **+** | **+** | **+** | **-** | **+** |
| ***S.* Enteritidis D24954** | **+** | **-** | **-** | **+** | **+** |
| ***S.* Senftenberg 20050439** | **+** | **-** | **-** | **-** | **-** |
| ***S.* Enteritidis SL7488** | **+** | **+** | **-** | **-** | **+** |
| ***S.* Agona 20071186** | **-** | **+** | **-** | **-** | **+** |
