# Supplementary material for: Bactericidal Immunity to Salmonella in Africans and Mechanisms Causing Its Failure in HIV Infection
Source: PLoS Negl Trop Dis. 2016 Apr 8;10(4):e0004604. doi: 10.1371/journal.pntd.0004604 (PMC4825999; doi:10.1371/journal.pntd.0004604)
Supplement: S3 Table — (DOCX) [file pntd.0004604.s003.docx]

**S3 Table.** **Anti-*S*. Typhimurium LPS antibody concentrations in undiluted sera and in purified antibody isotype fractions.**

|  |  | **Anti-LPS antibodies in 1 ml undiluted sera (mg)** | | |  | **Anti-LPS antibodies in 1 mg of total purified isotype fraction (mg)** | | |
| --- | --- | --- | --- | --- | --- | --- | --- | --- |
|  | **Sera #** | **IgA** | **IgG** | **IgM** |  | **IgA** | **IgG** | **IgM** |
| **HIV- control** | **1** | 0.028 | 0.319 | 0.032 |  | 0.015 | 0.171 | 0.068 |
|  | **2** | 0.076 | 0.343 | 0.033 |  | 0.044 | 0.175 | 0.035 |
|  | **3** | 0.036 | 0.367 | 0.029 |  | 0.022 | 0.162 | 0.031 |
|  |  |  |  |  |  |  |  |  |
| **HIV+ killing** | **1** | 0.022 | 0.352 | 0.023 |  | 0.017 | 0.067 | 0.028 |
|  | **2** | 0.023 | 0.375 | 0.035 |  | 0.014 | 0.082 | 0.036 |
|  | **3** | 0.056 | 0.394 | 0.026 |  | 0.012 | 0.085 | 0.037 |
|  | **4** | 0.037 | 0.313 | 0.032 |  | 0.007 | 0.067 | 0.031 |
| **HIV+ inhibitory** |  | 0.794 | 1.566 | 0.093 |  | 0.277 | 0.215 | 0.106 |
|  | **1** |  |  |  |  |  |  |  |
|  | **2** | 0.340 | 1.004 | 0.090 |  | 0.058 | 0.238 | 0.037 |
|  | **3** | 0.360 | 0.821 | 0.028 |  | 0.087 | 0.171 | 0.033 |
|  | **4** | 0.541 | 0.744 | 0.023 |  | 0.087 | 0.168 | 0.046 |
|  | **5** | 0.648 | 0.768 | 0.023 |  | 0.261 | 0.175 | 0.030 |
